# The influence of mentalization and psychological flexibility on the mental health of graduate students in China: a cross-sectional study

**DOI:** 10.3389/fpsyt.2025.1482079

**Published:** 2025-07-07

**Authors:** Xinghua Wang, Xiaosha Yang, Xuemei Li

**Affiliations:** ^1^ Department of Psychiatry, Hechuan District of Chongqing Mental Health Center, Chongqing, China; ^2^ Department of Psychiatry, The First Affiliated Hospital of Chongqing Medical University, Chongqing, China

**Keywords:** mentalization, mental health, psychological flexibility, graduate students, GAD-7, PHQ-9

## Abstract

**Background:**

Psychological health problem has become an important health problem of graduate students worldwide. Mentalization and mental flexibility are important factors related to mental health, but their relationship has not been discussed before in graduate students. The purpose of this study was to evaluate the mental health status of postgraduates and to determine whether mentalization and psychological flexibility are significant factors affecting the mental health status of postgraduates.

**Methods:**

samples of the communist party of China, there are 2728 graduate students. GAD-7, PHQ-9 were used to assess mental health. Mind through mental questionnaire (MZQ) measure, the Acceptance and Action Questionnaire 2nd Edition (AAQ-II) and Cognitive Fusion Questionnaire (CFQ-F) to evaluate mental flexibility. Multiple factors analysis and linear regression analysis is used to determine the Mentalization, the mental flexibility and the relationship between the participants’ mental health.

**Results:**

There were significant differences in the scores of anxiety and depression in gender, residence, left-behind experience, and parents’ marital status. The scores of the four dimensions of mentalization were positively correlated with the scores of anxiety. In mentalization, MZQ-refusing self-reflection, MZQ-emotional awareness, MZQ-emotional regulation score and depression score were positively correlated. Psychological flexibility played a partial mediating role in the four dimensions of mentalization and anxiety scores. The mediating effect of cognitive fusion was not clear in the four dimensions of mentalization and depression scores. Acceptance and action played a partial mediating role in refusing self-reflection, emotional awareness, and regulation of affect, as well as a full mediating role in psychic equivalence mode and depression scores.

**Conclusions:**

Mentalization and psychological flexibility are significantly correlated with anxiety and depression in graduate students. The four dimensions of mentalization and psychological flexibility exhibit varying degrees and linear relationships with graduate students’ anxiety and depression. In the relationship between mentalization and anxiety-depressive emotions, the mediating effects of acceptance and behavior, as well as cognitive fusion, are different. This suggests that investigating mentalization and psychological flexibility in relation to anxiety and depression is meaningful. Mentalization and psychological flexibility may potentially serve as important indicators of graduate students’ mental health in the future.

## Introduction

Mental health is an important indicator of health (1). Mental health problems have become a major public health problem worldwide because of their increasing prevalence and heavy burden on families or society (2, 3). Suicide prevention is also a global priority (4). Graduate students, for their part, the mental health challenges facing bigger, studies have shown that graduate students are under a lot of stress and anxiety, in a study on the southeast of a large university graduate student’s mental health study, found that 7.3% of the samples have suicidal thoughts, said 2.3% of the sample has a suicide plan, 1.7% of the sample reported harming themselves in the past 2 weeks, while 9.9% reported having attempted suicide in their lifetime (5). The main causes of suicide included graduation pressure, depression and academic pressure. Most suicides occurred in graduation years, and the main methods of suicide were jumping off buildings and hanging (6). Graduate students mental health problems is not only the problems facing in our country, a study shows 34.8% of the world’s graduate students suffering from anxiety disorders, including mild anxiety (19.1%), moderate anxiety (15.1%), severe anxiety (10.3%), and the anxiety disorder prevalence is on the rise since 2005 (7). Meta-analysis also showed that the pooled prevalence of overall, mild, moderate and severe depressive symptoms in graduate students was 34%, 27%, 13% and 8%, respectively, suggesting that graduate students are at high risk of depression (8). Early identification and management of psychological problems is of great significance for graduate students.

Mentalization is a concept proposed by Bateman, Fonagy and colleagues to better understand people with borderline personality disorder (BPD) (9). In group and individual psychotherapy for BPD, the focus of treatment is on increasing the patient’s capacity for mentalization, which refers to the ability to consider one’s own and others’ mental states as distinct yet potentially causing actions. The conclusion drawn from the research suggests that the core aspects of treating BPD include enhancing mentalization, taking into account the patient’s deficits, utilizing transference, maintaining psychological closeness, and working with current mental states. Finally, it is proposed that mentalization serves as a common theme in the psychotherapy of BPD, which may explain why different treatments are considered “effective.” Mentalization capacity can be conceptualized as a mental process that involves thinking about one’s own and others’ thoughts, needs, feelings, wishes, and desires. The ability to understand and interpret the mental states (e.g. emotions, beliefs, intentions, etc.) underlying the actions of oneself and others (10, 11). Mentalization plays an important role in emotion regulation, social interaction and mental health. Mentalization affects the individual’s emotional experience and expression ability, is significantly related to mental health, and is closely related to personality development (12–14). Mentalization seems to manifest differently in different treatments, and the mentalization capacity of patients seems to be related to the psychotherapy process (15). Psychological change is more is not a single phenomenon: its four dimensions: refusing self-reflection, emotional awareness, psychic equivalence model and regulation of affect, differences associated with clinical symptoms of mental illness (16). In response to the continuous development of mentalization, “mentalization based therapy” (MBT) was developed, and mentalization has since been associated not only with borderline personality disorder (BPD), but also with depressive mood and so on (12, 17–19). Mentalization-based therapy for adolescents (MBT-A), an evidence-based intervention that has shown effectiveness in helping young people with self-harm, borderline personality, and depression. As such, mentalizing can be viewed as the property of all good psychotherapy (20). All these studies suggest that the mental effect on mood has important significance.

Psychological flexibility is a fundamental aspect of mental health (21). Psychological flexibility is the core concept of ACT, which refers to the flexibility of individuals to accept their inner experiences and take actions consistent with their own values in the face of various psychological and emotional challenges (22). ACT differs from traditional cognitive behavioral therapy (CBT) in that ACT tends to promote acceptance, whereas CBT focuses on changing maladaptive attitudes (23). In the acceptance and Commitment therapy (ACT) model, psychological inflexibility is considered to be the main source of distress, and the central goal of ACT is to improve psychological flexibility (24). Empirical evidence for clinical outcomes in anxiety, depression, and post-traumatic stress disorder (PTSD) is satisfactory (25). Another study explored the relationship between psychological flexibility and academic mood. The results showed that higher psychological flexibility was positively associated with positive affect and success expectations, and negatively associated with task avoidance and negative affect. This suggests that psychological flexibility not only contributes to learning success but also improves emotional management in students (26). A wide range of research is reviewed showing that many forms of psychopathology can be conceptualized as unhealthy efforts to escape and avoid emotions, thoughts, memories, and other private experiences (27). Acceptance and behavior become the psychological problems and risk factors for lower quality of life (21, 28). Therefore, it can be seen that mentalization, psychological flexibility and mood are clearly correlated, and it can be inferred that the improvement of psychological flexibility can also help the improvement of mentalization.

To sum up, both mentalization and psychological flexibility play important roles in mental health, but there is no research on the relationship between mentalization and psychological flexibility in mental health problems. The mentalization, the psychological flexibility in different psychological cornerstone of therapy plays an important role, but they work together in the relationship between mental health problems is unclear. And the mentalization, the psychological flexibility in the graduate of this particular crowd psychological health problem is not yet clear. The mentalization level and psychological flexibility both play significant roles in mental health. Further exploring the extent of their contributions and the potential linear relationship between them is of great importance.

This study will explore the relationship between mentalization and psychological flexibility through the mental health status of graduate students, and explore the correlation between mentalization, psychological flexibility and graduate students’ emotions. Therefore this study need to clear the graduate student’s psychological health status, and analyze the mental and psychological flexibility correlation with graduate students sentiment. This study is expected to provide valuable information in the graduate student’s mental health services.

## Methods

### Subjects and study design

This research take the convenience sampling method, choose a full-time colleges and universities of Chongqing, adopt the method of network questionnaire survey, 17217 questionnaires taken back towards the school students, is a graduate students of 2729. After excluding questionnaires with at least one missing value for participants, we obtained 2,728 valid questionnaires from graduate students. Prior to assessment, all subjects were informed about the purpose of the study, the confidentiality of personal information, the voluntary principle, and all participants gave informed consent prior to participation. Materials and analysis code for this study are available by emailing the corresponding author. Study protocols were approved by the ethics committee of Chongqing Medical University, China.

### Measures taken

#### Population statistics

General information including age, gender, educational background, of seat of registered permanent residence (city, town, remote rural), whether the one-child, whether to stay experience, parents’ marital status.

#### Mentalization

Mind using mental questionnaire (MZQ) measurement, MZQ original version is developed by Hausberg etc (29), is a kind of psychological evaluation from patients with Angle of the self-assessment tool. It contains 15 items with responses ranging from 1 = “not at all” to 5 = “entirely” on a 5-point scale. It is divided into four subscales with acceptable reliability and adequate validity. Subscales included “refusing self-reflection” (4 items), “emotional awareness” (4 items), “psychic equivalence mode” (4 items), and “regulation of affect” (3 items). The score of each subscale was the average of the sum of the scores of each item, and the total score of the scale was the average of the sum of the scores of 15 items. The higher the calculated score of the total scale, the lower the psychological ability. The Cronbach’s alpha coefficient of the Center Intelligence questionnaire (MZQ) was 0.934.

#### Psychological flexibility

Psychological flexibility with the Chinese version of acceptance and action questionnaire second edition (AAQ - II) and cognitive fusion questionnaire (CFQ - F) measurements. The CFQ-F, which measures cognitive fusion, is a 9-item self-report questionnaire on a 7-point scale with total scores ranging from 9 to 63, with higher scores indicating greater psychological rigidity. AAQ-II measures experiential avoidance as a 7-item self-report questionnaire on a 7-point scale with total scores ranging from 7 to 49, with higher scores indicating greater psychological rigidity.The Cronbach’s alpha coefficients of the CFQ-F and AAQ-II questionnaires in this study were 0.962 and 0.952, respectively.

#### Mental health

Mental health was measured by Patient Health Questionnaire-9 (PHQ-9) and 7-item Anxiety Scale (GAD-7).

Patient Health Questionnaire-9 (PHQ-9) is a scale for screening depression, which is widely used in clinical practice and research. The PHQ-9 has good validity and reliability in identifying depression and assessing its severity (30). The PHQ-9 is not only effective in screening for depression in different medical Settings (31), but also applicable to screening for depression in the general population (32). The PHQ-9 is a self-testing instrument consisting of a 9-item self-report questionnaire. Using a 4-point scale, as a measure of severity, each of the nine items can be scored from 0 (not at all) to 3 (almost every day), with total scores ranging from 0 to 27, with higher scores indicating greater depression. In this study (PHQ - 9) questionnaire Cronbach ‘s alpha coefficient is 0.898.

The 7-item Anxiety Disorder-7 (GAD-7) has good validity and reliability in identifying generalized anxiety disorder and assessing its severity (33). GAD-7 is not only suitable for clinical patients, but also suitable for screening anxiety disorders in the general population (34). GAD - 7 anxiety is a self-report questionnaire, including 7 items in a questionnaire. A 4-point scale was used, as a measure of severity, with a total score ranging from 0 to 21 (higher scores indicate more severe symptoms). The Cronbach’s alpha coefficient of GAD-7 questionnaire in this study was 0.921.

### Statistical analysis

IBM SPSS Statistics 27.0 was used for data analysis. Descriptive statistics were performed for study variables and general characteristics. Univariate analyses were used to determine the relationship between sociodemographic characteristics, mentalization, psychological flexibility, and mental health. In addition, linear regression analyses were constructed to analyze the relationship between mental health and mentalization and psychological flexibility. The mediating effect of psychological flexibility on mentalization and mental health was explored using the Bootstrap procedure. P (two-tailed) < 0.05 was considered statistically significant.

## Results

Recycling effective questionnaire 2728, for the next step analysis. The subjects were between 19 and 41 years old, with an average age of 24.37 years (SD=1.92), and 69.7% of them were males. The proportion of city, town, remote rural was 25.3%, 37.2% and 37.6%, respectively. 57.2% had more than one sibling, most of them had not experienced left-behind (n=1837; 67.3%), the parents’ marital status is duration of majority (n = 2457; 90.1%) (**Table 1**).

**Table 1:** Study sample description (N = 2728).

Sociodemographic	Total (N=2728)
Age (years), Mean (SD)	24.37 ± 1.92
Residence, n (%)	
city	689(25.3%)
town	1014(37.2%)
remote rural	1025(37.6%)
Gender, n (%)	
males	1901(69.7%)
females	827(30.3%)
Being the only child or not, n (%)	
yes	1168(42.8%)
no	1560(57.2%)
Experience of left-behind, n (%)	
yes	891(32.7%)
no	1837(67.3%)
Parental marital status, n (%)	
survival	2457(90.1%)
divorced	195(7.1%)
widowed	76(2.8%)

As shown in **Table 2** of our respondents, the average total score of MZQ scale is 1.736, refusing self-reflection score is 1.695, emotional awareness score is 1.680, psychic equivalence mode score is 1.851, and regulation of affect score is: 1.711, which shows that the mentalization ability of graduate students is higher in this survey; The average score of CFQ-F cognitive fusion was 25.390, and the average score of AAQ-II acceptance and action was 15.680. According to the scoring rules, the degree of thought rigidity of graduate students was medium to low.

**Table 2:** Distribution of PHQ-9, GAD-7, MZQ, AAQ-II, and CFQ-F (N = 2728).

	Mini value	Max value	Mean value	SD
GAD-7	0	21	1.970	3.060
PHQ-9	0	27	2.010	3.312
Total score of MZQ	1	5	1.736	0.742
refusing self-reflection	1	5	1.695	0.783
emotional awareness	1	5	1.680	0.815
psychic equivalence mode	1	5	1.851	0.865
regulation of affect	1	5	1.711	0.788
CFQ-F	9	63	25.390	12.685
AAQ-II	7	49	15.80	7.736

The average score of GAD-7 was 1.97. According to the scoring rules, GAD-7 was further divided into four grades (normative, mild, moderate and severe), of which 2212 (81.1%) had no anxiety. The average score of PHQ-9 was 2.01, and according to its scoring rules, it was further divided into 5 grades (normative, mild, moderate, moderate to severe, severe). Among them, 2275 people were not depressed, accounting for 83.4% (**Table 3**).

**Table 3:** Distribution of GAD-7 and PHQ-9 scores (N = 2728).

		Graduate students (N=2728)	
	Grades	n	n (%)
GAD-7	normative	2212	81.1	
	mild	452	16.6	
	moderate	46	1.7	
	severe	18	0.7	
PHQ-9	normative	2275	83.4	
	mild	366	13.4	
	moderate	56	2.1	
	moderate to severe	23	0.8	
	severe	8	0.3	

**Table 4** shows that mentalization, cognitive fusion, acceptance and action were positively correlated with anxiety and depression (P<0.001). **Table 4** also showed that gender and left-behind experience were related to anxiety (P <0.05), and residence, left-behind experience, and parents’ marital status were related to depression (P <0.05).Residence, gender, left-behind experience, and parents’ marital status were not strictly according to the control study, which may have certain errors in the research results.

**Table 4:** Influence factors of anxiety and depression (mean ± SD).

Sociodemographic	GAD-7	r/t/F	P	PHQ-9	r/t/F	P
Age (years)		-0.024	0.203c		-0.019	0.314c
Gender		-3.025	**0.003a**		-1.726	0.084a
males	1.86 ± 3.162			1.94 ± 3.452		
females	2.24 ± 2.795			2.18 ± 2.959		
Residence		2.354	0.095b		4.007	**0.018b**
city	1.76 ± 2.732			1.73 ± 2.819		
town	2.05 ± 3.003			2.19 ± 3.450		
remote rural	2.05 ± 3.309			2.03 ± 3.465		
Being the only child or not		-1.043	0.297a		-0.32	0.746a
yes	1.90 ± 3.092			1.99 ± 3.443		
no	2.03 ± 3.036			2.03 ± 3.210		
Experience of left-behind		3.548	<0.001a		2.002	0.045a
yes	2.28 ± 3.277			2.20 ± 3.346		
no	1.82 ± 2.939			1.93 ± 3.292		
Parental marital status		2.958	0.052b		3.894	**0.020b**
survival	1.93 ± 3.044			1.96 ± 3.292		
divorced	2.45 ± 3.209			2.65 ± 3.668		
widowed	2.25 ± 3.116			1.97 ± 2.814		
Total score of MZQ		0.578	**<0.001c**		0.574	**<0.001c**
refusing self-reflection		0.521	**<0.001c**		0.527	**<0.001c**
emotional awareness		0.521	**<0.001c**		0.531	**<0.001c**
psychic equivalence mode		0.527	**<0.001c**		0.512	**<0.001c**
regulation of affect		0.540	**<0.001c**		0.523	**<0.001c**
CFQ-F		0.516	**<0.001c**		0.491	**<0.001c**
AAQ-II		0.543	**<0.001c**		0.525	**<0.001c**

at-value, bF-value, rc-value.The bold values indicate p<0.05.

**Table 5** shows that mentalization, cognitive fusion, and acceptance were positively correlated with action (P<0.001).

**Table 5:** Correlation analysis between mentalization and psychological flexibility.

		CFQ-F	AAQ-II
Total score of MZQ	r	0.704**	0.704**
	p	<0.001	<0.001
refusing self-reflection	r	0.632**	0.644**
	p	<0.001	<0.001
emotional awareness	r	0.641**	0.649**
	p	<0.001	<0.001
psychic equivalence mode	r	0.664**	0.638**
	p	<0.001	<0.001
regulation of affect	r	0.621**	0.631**
	p	<0.001	<0.001

** At the 0.01 level (two-tailed), the correlation is significant.

We treat mentalization and psychological flexibility as independent variables and the scores of anxiety and depression as dependent variables, resulting in the following relationship(**Table 6**). Linear regression analysis showed that further MZQ - refusing self-reflection and anxiety (B = 0.254, beta = 0.065, P = 0.033) and depression (B = 0.437, beta = 0.103, P < 0.001). MZQ - emotional awareness and anxiety (B = 0.327, Beta = 0.087, P = 0.003) and depression (B = 0.576, beta = 0.142, P < 0.001). MZQ - psychic equivalence mode and anxiety (B = 0.246, beta = 0.070, P = 0.019) and depression (B = 0.138, Beta = 0.036, P = 0.228). MZQ - regulation of affect and anxiety (B = 0.722, beta = 0.185, P < 0.001) and depression (B = 0.657, beta = 0.156, P < 0.001). The cognitive fusion and anxiety (B = 0.020, β = 0.217, P= 0.002), depression (B =0.013, β = 0.049, P=0.069). The acceptance and action and anxiety (B =0.086, β = 0.217, P <0.001), depression (B =0.089, β = 0.208, P <0.001), P<0.001). MZQ-psychic equivalence mode, cognitive fusion may have a non-linear relationship with depressive emotions, rather than a simple linear relationship.

**Table 6:** Linear regression of the mentalization, the psychological flexibility with anxiety and depression.

Independent variable	GAD-7			PHQ-9		
	B	95%CL		B	95%CL	
		Lower	Upper		Lower	Upper
	-2.522			-3.143		
refusing self-reflection	0.254	0.021	0.487	0.437	0.182	0.692
emotional awareness	0.327	0.114	0.540	0.576	0.343	0.809
psychic equivalence mode	0.246	0.041	0.451	0.138	-0.086	0.362
regulation of affect	0.722	0.523	0.920	0.657	0.440	0.874
CFQ-F	0.020	0.007	0.033	0.013	-0.001	0.027
AAQ-II	0.086	0.065	0.106	0.089	0.066	0.112

after adjusted for gender, place of residence, left-behind experience, and parental marital status.

In order to further understand the mind, the mental flexibility, a graduate students of the relationship between anxiety and depression, explore the psychological flexibility in mind whether the mediation role between anxiety and depression. First of all, to standardization of prediction variables, anxiety depression score as the dependent variable, select the four dimensions of mental scores as prediction variables, at the same time choose AQQ - II scores and CFQ - F score as intermediary variable intermediary effect inspection, the bootstrap sampling frequency set to 5000 times. In the score of anxiety, it was found that psychological flexibility played a partial mediating role in the four dimensions of mentalization. In refusing self-reflection as a predictor variable, the mediating effect value was 0.171 (cognitive fusion 0.034, acceptance and action 0.136).In emotional awareness as prediction variables, intermediary effect value of 0.262 (0.192) 0.071 cognitive fusion, acceptance and action; As a predictor variable in psychic equivalence mode, the mediating effect value was 0.208 (cognitive fusion 0.088, acceptance and action 0.120).In regulation of affect as a predictor variable, intermediary effect value of 0.238 (0.181) 0.056 cognitive fusion, acceptance and action; In depression score, psychological flexibility in the mediation of cognitive fusion effect is not obvious (effect of 0), Acceptance and action played a partial mediating role in the three dimensions of refusing self-reflection, emotional awareness, and regulation of affect. Acceptance and action played a complete mediating role in psychic equivalence mode(effect size was 100%). As in **Table 7**, Cognitive fusion does not intermediary effect between mentalization and depressive affect, and the MZQ-psychic equivalence mode is not direct effect to depressive affect. This is consistent with the absence of a linear relationship between cognitive fusion, MZQ-psychic equivalence mode, and depressive affect (**Table 6**).

**Table 7:** The mediating role of psychological flexibility between mentalization and anxiety and depression.

		GAD-7				PHQ-9			
		Value	95% CI		Relative effect proportion%	Value	95% CI		Relative effect proportion%
			Lower	Upper			Lower	Upper	
refusing self-reflection	Total effect	0.404**	0.167	0.642	100	0.586**	0.328	0.844	100
	Direct effect	0.234*	0.002	0.466	57.83	0.424**	0.17	0.678	76.06
	indirect effect	0.171	0.026	0.063	42.17	0.162	0.023	0.057	23.94
	CFQ-F	0.034	0.003	0.021	8.52	0.022	0	0.015	0
	AAQ-II	0.136	0.02	0.054	33.66	0.14	0.019	0.052	23.94
emotional awareness	Total effect	0.584**	0.37	0.799	100	0.818**	0.585	1.051	100
	Direct effect	0.322**	0.11	0.534	55.1	0.575**	0.343	0.807	75.85
	indirect effect	0.262	0.05	0.093	44.9	0.243	0.042	0.081	24.15
	CFQ-F	0.071	0.006	0.037	12.1	0.045	-0.001	0.027	0
	AAQ-II	0.192	0.032	0.074	32.8	0.198	0.03	0.071	24.15
psychic equivalence mode	Total effect	0.470**	0.265	0.675	100	0.328**	0.105	0.551	100
	Direct effect	0.262*	0.058	0.466	55.71	0.147	-0.076	0.371	0
	indirect effect	0.208	0.039	0.08	44.29	0.181	0.029	0.068	100
	CFQ-F	0.088	0.007	0.046	18.68	0.056	-0.002	0.035	0
	AAQ-II	0.12	0.02	0.05	25.62	0.124	0.019	0.048	100
regulation of affect	Total effect	0.968**	0.768	1.168	100	0.882**	0.665	1.1	100
	Direct effect	0.730**	0.533	0.928	75.45	0.659**	0.443	0.876	78.82
	indirect effect	0.238	0.045	0.079	24.55	0.223	0.038	0.07	21.18
	CFQ-F	0.056	0.005	0.03	5.83	0.036	-0.001	0.022	0
	AAQ-II	0.181	0.031	0.065	18.72	0.187	0.029	0.062	21.18

* indicates p<0.05, ** indicates p<0.01.

## Discussion

To the best of our knowledge, this is the first study to investigate the correlation between the mental health of graduate students and their mentalization and psychological flexibility. Additionally, this is the first exploration of the mediating role of psychological flexibility between mentalization and anxiety and depression levels. The mediating effect of psychological flexibility between mentalization and mental health was further explored. PHQ-9 and GAD-7 are the most famous mental health screening scales in the world, which can maintain good consistency in different populations and can be used to screen mental health (35). In practical application, the PHQ-9 and GAD -7 has been used in many fields, such as general health, mental health care, and different people, such as college students, people with chronic diseases, etc. of screening and evaluation (36). In addition, these scales are simple to use and can quickly identify people who need further mental health assessment and intervention.

In this study, it was shown that 81.1% of graduate students did not have anxiety disorders. Mild anxiety was observed in 16.6% of graduate students. Moderate anxiety was found in 1.7% of graduate students, while severe anxiety was present in 0.7% of graduate students. 83.4% of graduate students did not have depressive disorders. Mild depression was observed in 13.4% of graduate students. Moderate depression was found in 2.2% of graduate students. Moderate to severe depression was present in 0.8% of graduate students, while severe depression was observed in 0.3% of graduate students. Our study found that 18.9% of graduate students may have anxiety disorders and 16.6% may have depression. The results are slightly lower than previous domestic and international research, but this does not rule out the possibility that differences in the screening scales could be responsible for the discrepancy. Nonetheless, it is evident that anxiety and depression have become significant mental health issues among graduate students that cannot be overlooked.

In a number of studies have showed that women are more likely than men to suffer from anxiety and depression (37), the gender differences in different ages and social backgrounds are widespread, for example, a study of adolescents in Belgium, found that women in anxiety and depression score is significantly higher than male (38). However, the present study indicates that there is a difference in anxiety scores based on gender, but no difference in depression scores. Among the studies on the relationship between place of residence and anxiety and depression, some studies suggest an association, such as Amoran O et al (39). In the Nigerian population, the incidence of depression in rural areas was higher than that in urban areas, while some studies suggest no significant association, such as Kasturi S et al (40). There was no significant difference in the prevalence of depression and anxiety between rural and urban Australian youth. While in this study, prompt settlement and depression scores have differences, but with no obvious difference was found between anxiety score. One study was about sustained effects of left-behind experience during childhood on mental health in Chinese university undergraduates, suggesting the left-behind students experience more psychotic symptoms, including depression, anxiety, somatoform disorder, obsessive-compulsive disorder and suicide attempts and deliberate self-harm (41). In the present study, the left-behind experience was significantly different for anxiety scores, but different for depression scores. Parental marital status has a significant impact on the mental health of children and adolescents (42). In this study, we found that there was a difference in depression scores based on the marital status of parents.

In conclusion, in this study, it can be seen that there are differences in anxiety and depression scores in terms of gender, place of residence, left-behind experience and parents’ marital status. Among them, gender and left-behind experience are significantly different from anxiety scores, while place of residence and parents’ marital status are different from depression scores.

In recent years, the graduate student mental health research shows the trend of increasing, people began to pay more attention to the mental health status of the graduate students (43). In addition to the increasing attention to the mental health of graduate students, more treatment methods and health strategies have been proposed to solve the problem of the gradual decline in the mental health of graduate students (44). The study of the intrinsic mentalization abilities of graduate students is not only related to Mentalization-Based Treatment (MBT) but also to their mental health (45). In this study, the Mentalization Questionnaire (MZQ) was introduced to evaluate four dimensions of mentalization among graduate students. The higher the score, the lower the mentalization ability. The scores for the four dimensions of mentalization are positively correlated with anxiety scores (**Table 4**), indicating that the lower the mentalization ability, the higher the anxiety score and the more severe the anxiety. In mentalization, refusing self-reflection, emotional awareness, and regulation of affect scores were positively correlated with depression scores (**Table 4**), suggesting that the lower the mentalization ability of these three dimensions, the higher the depression score and the more severe the depression degree, while psychic equivalence mode had no significant correlation with depression scores. In the correlation between mentalization and mental flexibility, it can be seen that both suggest a significant correlation.

In addition, studies have found that the higher the mental flexibility not only associated with the low level of psychiatric symptoms, and with better function and lower stress levels and higher happiness (46), in this study, graduate student mental flexibility was significantly associated with anxiety and depression scales, including cognitive fusion and depression score linear regression was not significant, probably due to the multiple linear regression, There is a high collinearity between the independent variables, which leads to an increase in the standard error of the regression coefficient, and then affects the significance test. The acceptance and positively related to the linear regression suggests action and depression score, prompt the harder mental flexibility, a higher degree of depression. This study suggests the psychological flexibility degree is higher, the higher the degree of anxiety and depression. Psychological flexibility may be as a graduate student anxiety depression as one of the risk factors.

Further bootstrap method showed that visible psychological flexibility played a partial mediating role in the four dimensions of mentalization and anxiety score, and the proportion of mediating effect of visible acceptance and action was higher than that of cognitive fusion. Cognitive fusion does not have an obvious mediating effect on the four dimensions of mentalization and depression scores. Acceptance and action play a partial mediating role in the MZQ dimensions of refusing self-reflection, emotional awareness, and regulation of affect with depression scores. Acceptance and action have a complete mediating effect between the MZQ psychic equivalence mode and depression scores. In conclusion, the higher the mental flexibility score, the higher the mentalization score, and the higher the anxiety and depression score, suggesting that the more serious the mental rigidity and the lower the mentalization ability, the more serious the anxiety and depression. All these support that psychological flexibility and mentalization are closely related to mental health, and by improving psychological flexibility and mentalization, it is helpful to the recovery of psychological problems. Research has thoroughly demonstrated the impact of mentalization and psychological flexibility on mental health. Perhaps these factors are not only necessary to consider in the treatment of psychological problems but may also become important tools for assessing the severity of a patient’s condition.

Our study has some limitations. First, this study measured mentalization only using the Mentalization Questionnaire (MZQ) and measured psychological flexibility only using the Acceptance and Action Questionnaire II (AAQ-II) and the Cognitive Fusion Questionnaire (CFQ-F).In future research, it is advisable to use multidimensional measurement tools as much as possible to comprehensively reflect the participants’ psychological flexibility and mentalization. Secondly, the subjects of this study were all from the same university, so our results may better reflect the mentalization, mental flexibility and mental health of graduate students in a region. Finally, this study is a cross-sectional study, which cannot prove the causal relationship between variables. Therefore, in the follow-up study, longitudinal follow-up can be conducted to verify the relationship between mentalization, mental flexibility and mental health, and to verify the role of mental flexibility as a mediator between mentalization and mental health.

## Data Availability

The raw data supporting the conclusions of this article will be made available by the authors, without undue reservation.
